# Supplementary material for: An approach to optimizing dietary protein to growth and body composition in walking catfish, Clarias batrachus (Linneaeus, 1758)
Source: PLoS One. 2024 May 3;19(5):e0301712. doi: 10.1371/journal.pone.0301712 (PMC11068203; doi:10.1371/journal.pone.0301712)
Supplement: Attachment — Submitted filename: Response to Reviewers.docx [file pone.0301712.s001.docx]

Dear Sir,

The manuscript entitled “An approach to optimizing dietary protein to growth and body composition in walking catfish, *Clarias batrachus* (Linneaeus, 1758)” is improved/revised following the valuable suggestions of the reviewers.

All the corrections/changes are incorporated in the manuscript, and detailed point-to-point response is mentioned in the following table.

| **Sr** | **Reviewer Comment** | **Author Response** |
| --- | --- | --- |
| **Editor** | | |
| 1 | Ethics statement should only appear in the Methods section of your manuscript. If your ethics statement is written in any section besides the Methods, please move it to the Methods section and delete it from any other section. Please ensure that your ethics statement is included in your manuscript, as the ethics statement entered into the online submission form will not be published alongside your manuscript. | Added |
| 2 | In the online submission form, you indicated that  "The datasets used and analysed during the current study are available from the corresponding author on reasonable request.".  All PLOS journals now require all data underlying the findings described in their manuscript to be freely available to other researchers, either  1. In a public repository,  2. Within the manuscript itself, or  3. Uploaded as supplementary information. | All data underlying the findings described in their manuscript to be freely available to other researchers after acceptance of paper, either  1. In a public repository,  2. Within the manuscript itself, or  3. Uploaded as supplementary information |
| 3 | Please amend your list of authors on the manuscript to ensure that each author is linked to an affiliation.  We note that you have included affiliation numbers 1 and 2 however only affiliation1 have authors linked to them.  Please amend affiliation 2 to link an author to it or remove if added in error. | Author list amended. |
| 4 | 4. We are uncertain of our previous requests. To comply with PLOS ONE submissions requirements, in your Methods section, please provide additional information regarding the experiments involving animals and ensure you have included details on (1) methods of sacrifice, (2) methods of anesthesia and/or analgesia, and (3) efforts to alleviate suffering. | Added |
|  | **Reviewer 1** | |
| 1 | I would like to emphasize the importance of explicitly disclosing the dietary ingredients employed in the diet preparations outlined in this study. Additionally, it is crucial for the authors to clearly specify the protein source utilized in the study. This information is essential for ensuring transparency, reproducibility, and a comprehensive understanding of the experimental conditions. Please provide a detailed account of the dietary components and protein source to strengthen the integrity of the research. | Detailed dietary components of experimental feeds added as a new table (Table 1). |
| 2 | I suggest that the authors also include the proximate compositions of the experimental diets. Providing this information would add valuable detail to the study, aiding in the understanding of nutritional aspects and ensuring transparency for readers. | Proximate composition of the experimental diets is also added in Table 1. |
| 3 | It would be beneficial to provide the instrument names, manufacturers, and any relevant specifications in the Materials and Methods section. | Instrument names and manufacturersare added in the Materials and Methods section. |
| 4 | In line 113-114, the authors mention drying temp at 80ºC for water content analysis. However, it is essential to clarify the duration of this drying process. | Mentioned in the manuscript.  “Till constant of body weight” |
| 5 | I suggest the authors reconsider the use of the difference method for calculating protein content in the manuscript. While this method provides an approximate estimate, for more accurate and reliable results, it is advisable to employ established laboratory analysis method such as the Kjeldahl method. These methods are widely accepted for protein determination and would enhance the precision and credibility of the protein content reported in the study. | Difference method was used to evaluate the protein contents in this study. However, valuable suggestion of the reviewer will be considered for future studies. |
| 6 | I suggest that the authors consider investigating protein digestibility as part of this study. Including an analysis of protein digestibility would provide valuable insights into the bioavailability of dietary proteins and contribute to a more comprehensive understanding of the nutritional aspects under investigation. | Protein digestibility was not the part of this study. However, valuable suggestion of the reviewer will be considered for future studies. |
| 7 | On page 9, line 287, there appears to be a typo in the scientific name. It should be 'Ctenopharyngodon idella' instead of 'Ctenophyrngodon Idella.' Please correct this to ensure accuracy in the scientific nomenclature throughout the manuscript. | Scientific name is corrected. |
| 8 | There few typesetting errors throughout the manuscript that should be corrected accordingly | Typesetting errors throughout the manuscript are corrected. |
| **REVIEWER 2** | | |
| 1 | Insufficient novelty and significance. The researchers evaluate different crude protein percentages in the diets and assess their impact on factors such as weight gain, daily growth rate, protein efficiency ratio, and body composition, which are merely the most fundamental parameters used to evaluate feed formulations or to confirm the appropriate proportions of certain ingredients, and the work has already been done in closely related species. Since the experimental limitations and the lack of further investigation, a thorough and deep discussion could not be provided in the manuscript. Even the current data were not properly analyzed and discussed. It only indicated whether the results aligned or diverged from previous studies, without providing an explanation for the observed outcomes. To foster a more comprehensive discussion on this topic, additional research should be undertaken to explore factors such as enzyme activity, oxidative stress, gut microbiota, and blood physiological indicators, even immune response level. | Discussion is improved highlighting the novelty and significance of the study.  Explanation of observed outcomes of the study is added. |
| 2 | The introduction was not well written. No former studies about the function of crude proteins (or other nutritional ingredients) in upgrading biological traits in cultivated fish were introduced, which had already been widely studied and was supposed to be the most important information in this part. | Introduction is improved. |
| 3 | The authors should also provide more detailed information on the statistical methods used, as well as any potential limitations and sources of bias in their study. | Detail is added |
| 4 | Need thorough language editing. | Language is improved. |
| **REVIEWER 3** | | |
| 1 | The work is generally written in an understandable way, however there are several parts that are more confusing and deserve a grammatical and syntactic revision.  I report here a list of lines that should be reformulated to better clarify the concepts expressed: Lines 40-42; 60-61; 71-73; 200-206; 258-264. | Lines 40-42; 60-61; 71-73; 200-206; 258-264 are reformulated to make the statements more clear. |
| 2 | Important materials that must be provided in this manuscript concern: a complete table regarding the composition of the diets, with important emphasis on the origin (animal/vegetable) of the protein source used, and its amino acid composition. |  |
| 3 | Line 14: "Clarias batrachus is commercialy..." should be corrected as "Clarias batrachus is a commercially..." | Corrected as suggested by the reviewer. |
| 4 | Line 17: the symbol @ should be corrected with the symbol ≈ or ±. | @ is replaced with “at the rate of” |
| 5 | Line 31: "insignificant correlation" should be changed to "non-significant correlation". | “insignificant correlation" is changed to "non-significant correlation” |
| 6 | Line 47: the term "realized" is not appropriate, I suggest to use "considered" or "represent". | “realized” is replaced with "considered" |
| 7 | Line 53: "A vital issue" is not properly appropriate in this context, I suggest to change it. | "A vital issue" is replaced with “An important issue” |
| 8 | Line 68: "Henceforth" should be changed to "Therefore". | "Henceforth" is changed to "Therefore". |
| 9 | Lines 75-78: I suggest to reformualte the sentence in this way: "Fish proximate body composition constituents (fat, protein, water, organic content and ash) are influenced by diet, feed rate, sex, genetic strain, age, species and also by changing body size and condition factor [26-28]. | Corrected as suggested by the reviewer. |
| 10 | Line 93: the same correction reported for line 17. | @ is replaced with “at the rate of” |
| 11 | In material and methods should be also provided information regarding the illumination (light/dark cycle) during the trial. | Light/dark cycle during the trial is added in MM section. |
| 12 | Line 129: the same correction reported for line 31. | “insignificant correlation" is changed to "non-significant correlation” |
| 13 | Line 133: "Significant highest" should be changed to "Significantly highest". | "Significant highest" is changed to "Significantly highest". |
| 14 | Lines 150-152: I suggest to reformualte the sentence in this way: "Mean value of water, ash, fat and protein contents (% wet mass) were ranged from 74.10±0.31% to 79.23±0.52%, 3.12±0.07% - 4.68±0.05%, 3.90±0.06% - 4.43±0.05% and 13.09±0.58% - 16.79±0.27% in the studied treatments..." to avoid repetitions. | Reformulated as suggested by the reviewer. |
| 15 | Line 155: "and T6 than T4" should be changed to "and T6 than for T4". | "and T6 than T4" is changed to "and T6 than for T4". |
| 16 | Lines 201-202: I suggest to reformualte the sentence in this way: "the present experiment was found similar to that previously described by Tadesse [30]" to avoid repetitions. | Reformulated as suggested by the reviewer. |
| 17 | Line 207: "is a vital gauge". | Corrected by adding space. |
|  | Line 210: Although FCR value between 1.2 and 1.5 represent a good indicators, it could not be used as absolute standard in all fish species, as it can change according to several culturing factors. In addition the reference [33] reported this range in tilapia. I suggest to reformulate this sentence. | Reformulated.  Moreover, the following lines are added in Discussion for make it more clear:  Tadesse [30] reported the lowest feed conversion ratio (FCR=2.1) in fish fed with 40% CP, indicating its suitability for African catfish fingerlings than the other test diets. |
| 18 | Line 216: "animal ingredients" it should be clarified in M&M, providing further information about this source. | The line is reformulated to make clear.  Moreover, detailed dietary components of experimental feeds added as Table 1. |
| 19 | Line 281: there is a repetition of "in". | Corrected. |
|  | REFERENCES should be revised and checked accordingly to the journal requirements as I encountered some errors, such as Line 392, 400 and 416. | Corrected. |
| 20 | Table 1 and 2: It is not an error, however I suggest to substitute the title of the last column from "Sig." to "p-value", and the ".000" with "< .001". In Table 2 the caption is missing. | Table 1: "Sig." is substituted to "p-value", and the ".000" with "< .001".  Table 2: Footnote is added. |
| **REVIEWER 4** | | |
| 1 | Some sentences were not clear, you must formulate them correctly. Yet, English should be improved and reviewed by a native speaker. | Language of the manuscript is improved. |
| 2 | You should be precise about what factors might contribute to the 100% survival rate across all treatments. | Mentioned in the discussion section.  “…survival rate was recorded as 100% in all of the studied groups, indicating high tolerance of the fish in the confined system and also represents that rearing conditions were good (optimal).” |
| 3 | Could the study provide insights into why treatment with 40% crude protein (T4) resulted in the highest growth parameters for the studied specie? | It may be due to the high proportion of protein derived from easily digestible animal ingredients, as mentioned in discussion. |
| 4 | What implications do the variations in water, ash, fat, and protein content have for the nutritional value of C. batrachus in different treatments? | Results showed that feeding appropriate diet (containing 40% CP) to the fish resulted higher protein and lipid content in fish.  Thus following statement is added in the discussion:  Hence, the results indicated that farmer can achieve not only higher growth and low FCR but can attain higher protein and lipid contents in the *C. batrachus* by feeding the fish 40% crude protein, rather higher crude protein (45%CP) in diet. |
| 5 | How might the observed differences in body composition across treatments impact the quality of the fish as a food source? | As a higher proportion of protein content may exhibits the good quality of the fish as a food source. |
| 6 | Why did the treatment with 40% crude protein (T4) exhibit the best Feed Conversion Ratio (FCR)? Are there specific physiological or metabolic reasons for this result? | It may be due to the high proportion of protein derived from easily digestible animal ingredients, as mentioned in discussion. |
| 7 | How does body size affect the body composition of C. batrachus? | Described in the discussion section.  “Literature shows that body weight of a fish influences the various body constituents [16], and fat and protein increase while ash and water decrease with the increasing total length [56].” |
| 8 | In what ways does the condition factor correlate with body size, and why were these correlations mostly insignificant? | Mentioned in the discussion section.  “…condition factor, as body weight of a fish is not always proportional to the cube of its total length [60]” |
| 9 | How can the findings of this study be practically applied in aquaculture operations, especially in formulating optimal diets for C. batrachus? | Mentioned in the manuscript.  “Farmer can achieve not only higher growth and low FCR but can attain higher protein and lipid contents in the *C. batrachus* by feeding the fish 40% crude protein, rather higher crude protein (45%CP) in diet.”  “Data produced in the current research would be beneficial in evolving nutritionally balanced diets for the semi-intensive and intensive culture of this catfish.” |
| 10 | Are there any limitations or challenges in implementing the recommended 40% crude protein diet on a commercial scale? | Added in the discussion:  As, higher cost of a fish feed containing higher crude protein is considered a limitations or challenge in implementing the recommended 40% crude protein diet on a commercial scale, but farming industry should not compromise as it influences positively on the growth performance, FCR and quality of fish as food. |
