# Supplementary material for: An approach to optimizing dietary protein to growth and body composition in walking catfish, Clarias batrachus (Linneaeus, 1758)
Source: PLoS One. 2024 May 3;19(5):e0301712. doi: 10.1371/journal.pone.0301712 (PMC11068203; doi:10.1371/journal.pone.0301712)
Supplement: Attachment — Submitted filename: Response to Reviewer.docx [file pone.0301712.s002.docx]

Dear Sir,

The manuscript entitled “An approach to optimizing dietary protein to growth and body composition in walking catfish, *Clarias batrachus* (Linneaeus, 1758)” is improved/revised following the valuable suggestions of the reviewer.

All the corrections/changes are incorporated in the manuscript, and detailed point-to-point response is mentioned in the following table.

| **Sr** | **Reviewer Comment** | **Author Response** |
| --- | --- | --- |
| **REVIEWER 3** | | |
| 1 | Line 64-65: The correction made did not include "may BE higher". In addition, this part seems a bit confusiong, the author should clarify the concept better. FCR is a commonly used measure of conversion which is commonly used over the entire production life of a fish, and obviously, the amount of feed changes during this period, but this does not invalidate its importance. | Clarified the Line 64-65 |
| 2 | Line 107: The two new references 29 and 30 have incorrect numbers referring with the list of references, please check them. In addition, although accurate, the reference Preston et al.,2016, used for the calculation of proximate composition, does not include all the information for the correct evaluation of the ingredients listed in Table 1. Please provide further details or change the reference. | Numbering of references 29 and 30 are corrected.  Another reference is added for the calculation of proximate composition in Table 1. |
| 3 | Line 218: "feeding fish WITH 30%..." | Corrected Line 218 |
| 4 | Table 1. There is no indication about the unity of measurement of the ingredients. It must be indicated, together with the acronym CMC, in the caption. Furthermore, regarding the reported CP values, there are inconsistencies between the values and the name of the experimental diets for CP-35; CP-40; CP-45 and CP-50 (for these diets the effective amount of crude protein is lower). Since this part represents the core of the article and the author in the discussion and conclusion sections refers to the diet as the effective amount of crude protein, this information needs to be clarified and corrected. | Unit (%) added in the Table title.  Full form of CMC is added.  Format of treatment names made consistent and changed in discussion and conclusion section accordingly. |
